# Experimental data of the distillation of bio-oil from thermal cracking of methyl ester in castor oil

**DOI:** 10.1016/j.dib.2019.104325

**Published:** 2019-07-27

**Authors:** Guilherme Menshhein, Vanderlei Costa, Luana M. Chiarello, Dilamara R. Scharf, Edesio L. Simionato, Vanderleia Botton, Henry F. Meier, Vinicyus R. Wiggers, Laércio Ender

**Affiliations:** a Chemical Engineering Department, University of Blumenau, Blumenau, SC, Brazil; b Chemistry Department, University of Blumenau, Blumenau, SC, Brazil

**Keywords:** Distillation, Gas chromatographic analyses, Bio-oil

## Abstract

This article presents the experimental data on distillation of bio-oil obtained from thermal cracking of a mixture of castor oil and its methyl esters. The interpretation of the data can be found in Menshhein et al. (2019) available on https://doi.org/10.1016/j.renene.2019.04.136. Experiments were carried out using a simple distillation apparatus and the products were quantified and qualified from Gas Chromatography – Flame Ionization Detector (GC-FID) with standards compounds. Data were presented in terms of distillation equipment and distillation curve values of volume and temperature of the crude bio-oil sample. Information about GC-FID methods and chromatograms of from standard heptaldehyde and methyl undecenoate and their analytical curve. Carbon number data of crude bio-oil sample was also showed.

**Table:** Specifications table

Subject area	*Thermal cracking (pyrolysis) of triglycerides*
More specific subject area	*Bio-oil distillation*
Type of data	*Figures and tables*
How data was acquired	*Experiments, physicochemical and chromatographic analysis (distiller: B/R Instrument, model M690, GC-FID: 7890B/Agilent and 2010/Shimadzu)*
Data format	*Raw and tabulated data collection*
Experimental factors	*Volumetric data from distillation curve of thermal cracking fraction and chromatographic data*
Experimental features	*Distillation cuts of bio-oil from thermal cracking of methyl ester in castor oil*
Data source location	*Blumenau/SC – Brazil, University of Blumenau – FURB* *Chemical Engineering Department*
Data accessibility	*Data is with this article*
Related research article	*Menshhein* et al.*, Concentration of Renewable Products of Crude Bio-Oil from Thermal Cracking of the Methyl Esters in Castor Oil**[1]**.*

**Table:** 

**Value of the data** • The data provides details from noncomplex distillation equipment and the distillation curve of the bio-oil from thermal cracking of methyl ester in castor oil, which will enable comparison of results; • This data provides a GC-FID chromatogram and data from crude bio-oil presenting the different carbon range of this sample; • Information regarding GC-FID chromatograms detailing the analytical curves of heptaldehyde and methyl undecenoate standards.

## 1 Data

**Fig. 1:**
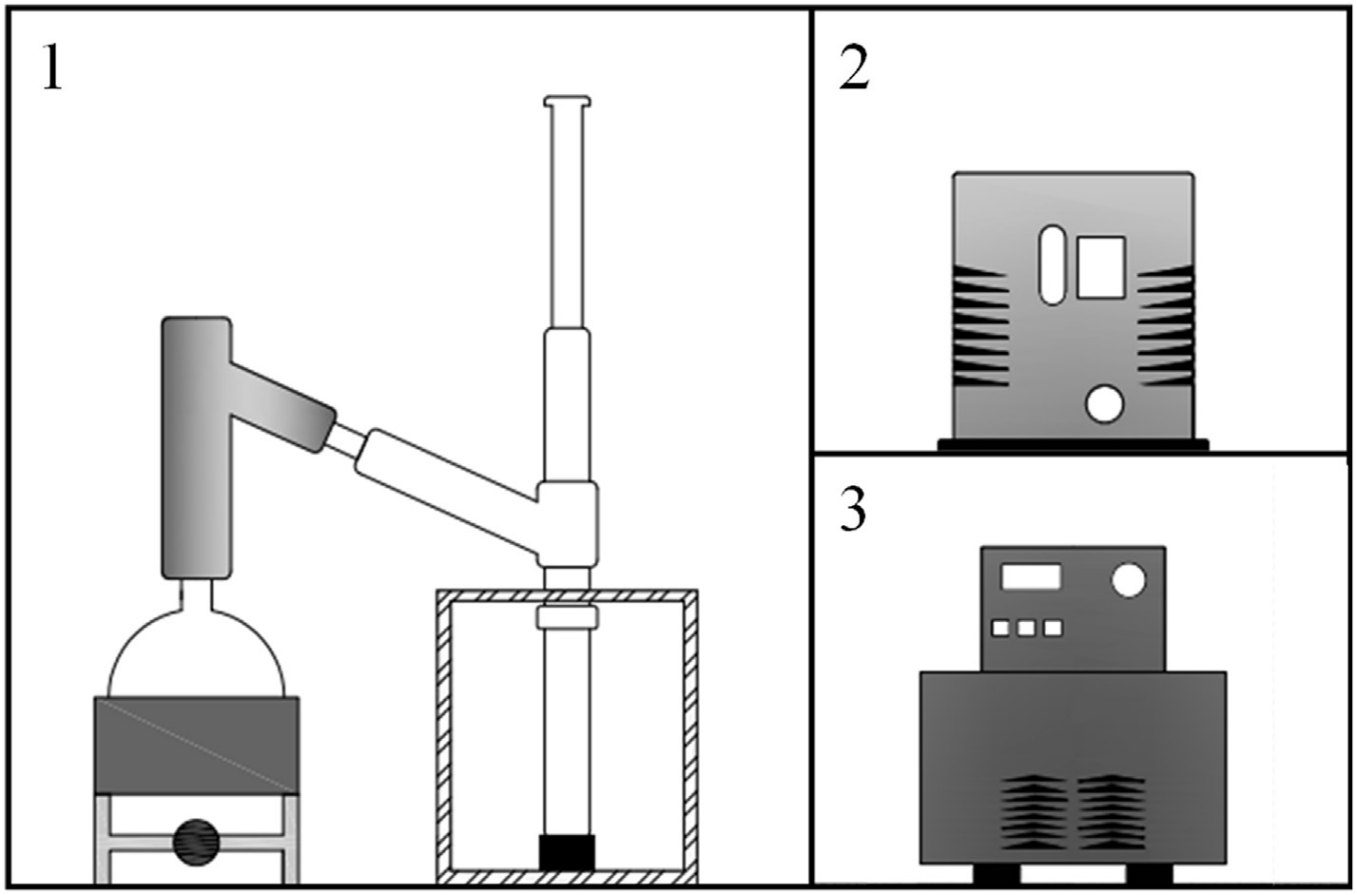
Distillation equipment design: 1) distillation apparatus 2) pump and 3) thermostatic bath.

**Fig. 2:**
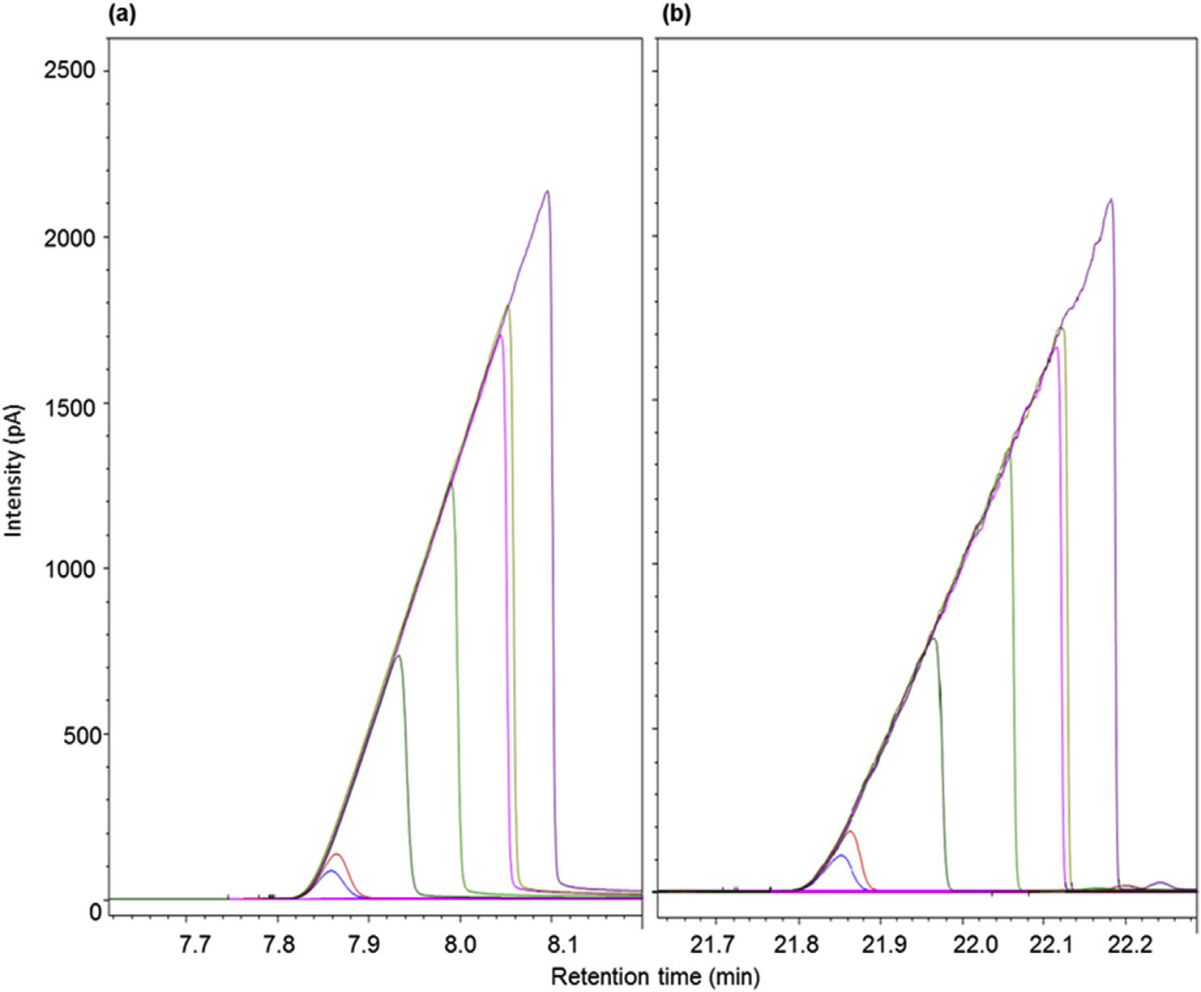
Chromatograms of GC-FID from standard of (a) heptaldehyde and (b) methyl undecenoate with concentration of these compounds varying from 0.46 to 48%.

**Fig. 3:**
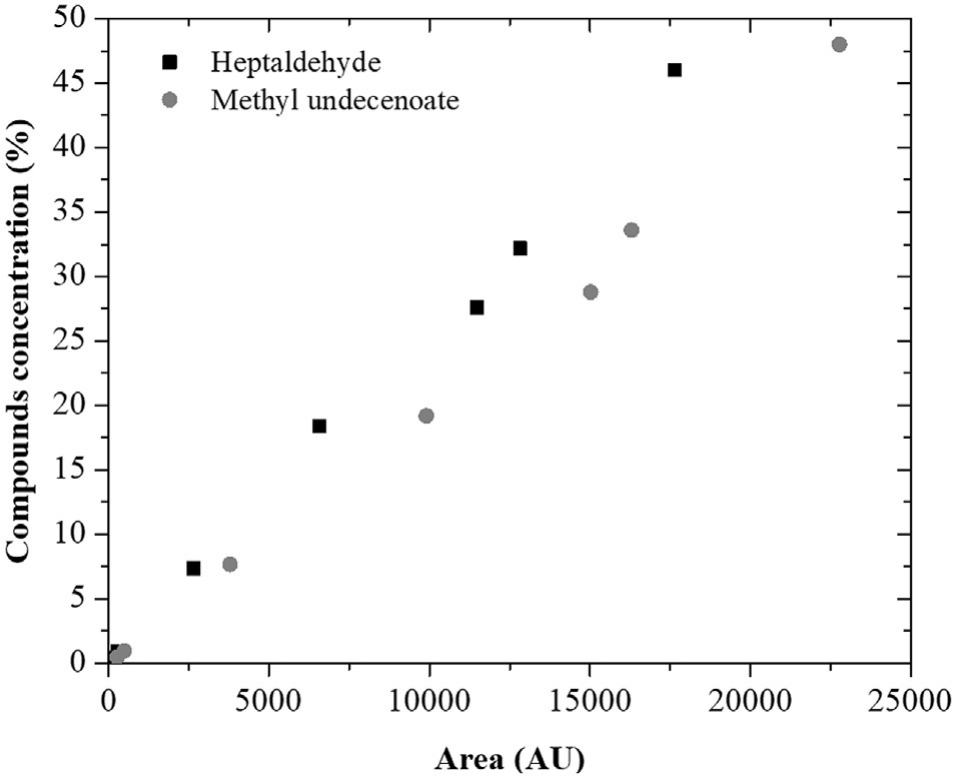
Analytical curve of heptaldehyde and methyl undecenoate, with R^2^ of 0.9962 and 0.9967, respectively.

**Fig. 4:**
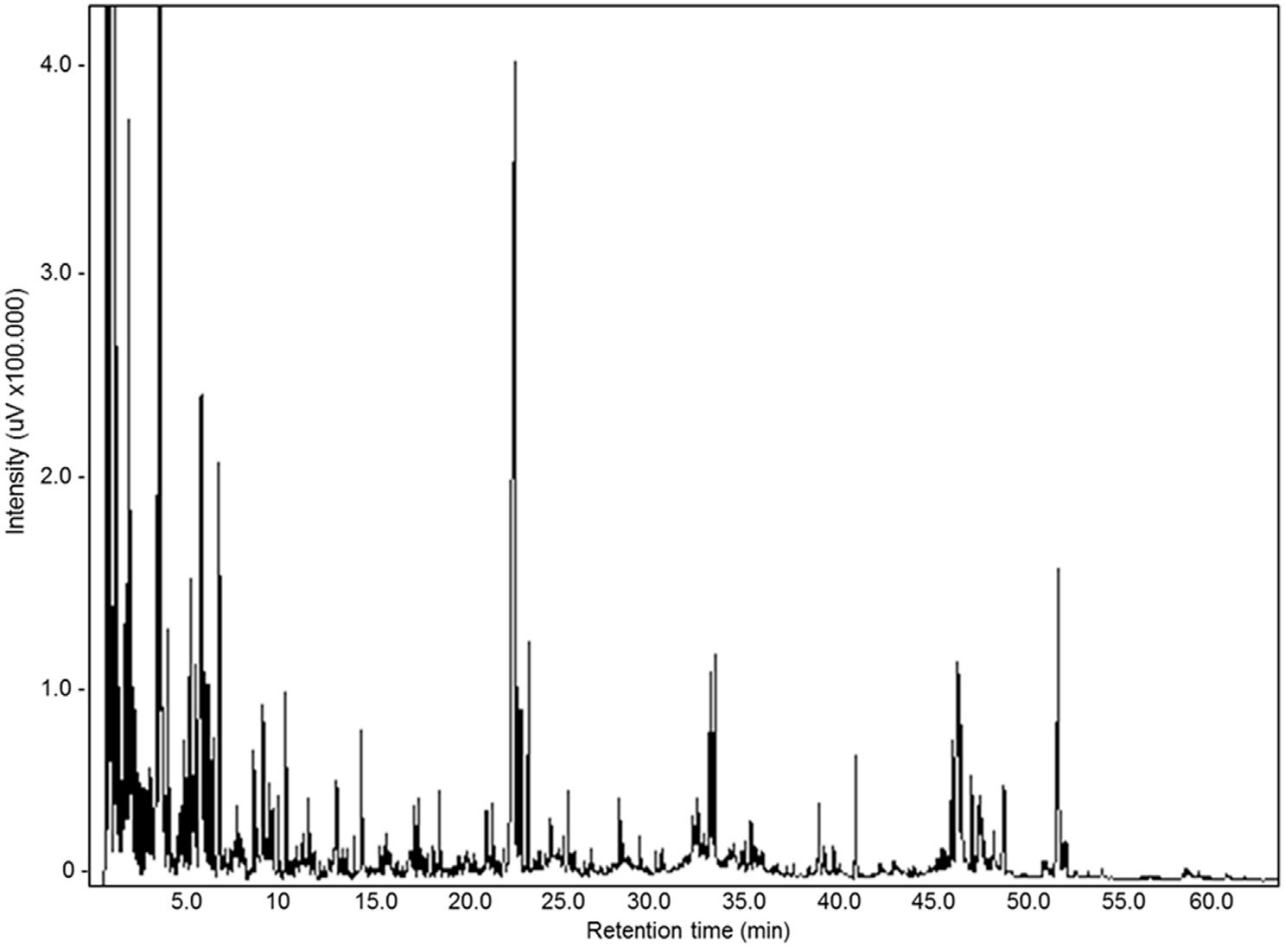
Carbon number chromatograms of crude bio-oil sample.

Fig. 1 presented the distillation equipment design. Table 1 shows the distillation curve values of volume and temperature of the crude bio-oil sample. Table 2 presents the gas chromatography methods used in this work. In Fig. 2, Fig. 3 and Table 3 are the observed chromatograms of GC-FID from standard heptaldehyde and methyl undecenoate and their analytical curve as graphic and table, respectively. Fig. 4 illustrates the chromatogram of carbon number of crude bio-oil sample. Table 4 presents the carbon number data for the crude bio-oil sample.

**Table 1:** Distillation curve data of the crude bio-oil sample.

Temperature (°C)	Distilled volume (%)
120.4 ± 4.3	0
144.0 ± 6.0	5
207.5 ± 5.4	15
216.6 ± 1.7	20
219.7 ± 1.2	25
221.5 ± 1.2	30
225.4 ± 5.8	40
235.3 ± 1.4	45
275.5 ± 3.9	50
297.9 ± 1.9	55
307.9 ± 7.3	60
314.6 ± 3.5	65
327.5 ± 7.9	70
348.6 ± 3.2	75
367.6 ± 4.3	80
378.1 ± 5.6	84
398.2 ± 7.9	85
412.3 ± 6.2	86
424.9 ± 1.2	87
439.2 ± 6.5	88

**Table 2:** Gas chromatography with flame ionization detector methods.

Description	Analyses		Column				Carrier gas				Oven Heating method					Injector/Detector T (°C)	
GC-2010 Shimadzu	Carbon number		OV-5 capillary column (30 m × 0.25 mm x 0.25 μm)				Helium				Initially 150 °C (for 1 min), increasing to 280 °C at a ramp of 5 °C min−1. The T was kept at 280 °C for 23 min.					250/280	
Standard	Heptaldehyde									Methyl undecenoate							
Purity (%)	≥92									96							
Supplier	Sigma-Aldrich									Sigma-Aldrich							
7890B Agilent	Desired compounds		Stabilwax capillary column (30 m × 0.25 mm x 0.25 μm)				Helium				Initially 50 °C (for 3 min), increasing to 250 °C at a ramp of 5 °C min−1. The T was kept at 250 °C for 7 min.					250/300	
Standard	C8	C9		C10	C11	C12		C13	C14			C15	C16	C17	C18		C19
Purity (%)	98.0	99.0		99.8	99.8	99.8		99.5	99.5			99.8	99.8	99.8	99.8		99.0
Supplier	S-A^a^	V^b^		F^c^	F	F		F	F			F	F	F	F		S-A

a S-A = Sigma-Aldrich.

b V = Vetec.

c F = Fluka.

**Table 3:** Data from analytical curve of heptaldehyde and methyl undecenoate.

Concentration (%)	Area (AU)	
	Heptaldehyde	Methyl undecenoate
0.46	185.2	–
0.48	–	272.5
0.92	286.3	–
0.96	–	492.1
7.36	2651.9	–
7.68	–	3789.8
18.4	6584.1	–
19.2	–	9906.2
27.6	11476.4	–
28.8	–	15029
32.2	12825.6	–
33.6	–	16298.2
46.0	17647.2	–
48.0	–	22780.7

**Table 4:** Carbon number range for the crude bio-oil sample.

Carbon Range	Crude bio-oil (%)
Below C9	14.79 ± 0.65
C9 to C10	41.16 ± 1.37
C10 to C11	5.31 ± 0.17
C11 to C12	3.87 ± 0.06
C12 to C13	2.07 ± 0.11
C13 to C14	2.16 ± 0.05
C14 to C15	12.65 ± 0.27
C15 to C16	1.09 ± 0.02
C16 to C17	1.50 ± 0.04
C17 to C18	3.65 ± 0.13
C18 to C19	0.79 ± 0.04
Above C19	11.13 ± 1.17

## 2 Experimental design, materials and methods

### 2.1 Materials

Experiments were carried out with bio-oil produced by Botton et al. [2] from thermal cracking of methyl ester in castor oil at 475–525 °C with residence time of 44–104 s.

### 2.2 Distillation curve

Experiments were performed in an automatic vacuum distiller as illustrated in Fig. 1 (B/R Instrument, model M690) [3], [4], based on the standards for petroleum characterization [5], [6]. The data obtained in this analysis is show in Table 1.

### 2.3 GC-FID analyses

All these analyses were performed in triplicate (Table 2). The desired compounds - heptaldehyde and methyl undecenoate - were analyzed by GC-FID using an Agilent GC-FID, model 7890B (Agilent Technologies, Inc., Wilmington, EUA) (Fig. 2, Fig. 3 and Table 3). The carbon number of bio-oil samples were analyzed using a Shimadzu GC-FID, according to Beims et al. [3] by n-alkane comparison (Fig. 4 and Table 4).
